# Rare cases of colonic schwannomas

**DOI:** 10.1093/jscr/rjac438

**Published:** 2023-12-29

**Authors:** Victor Gazivoda, Donghai Wang, Mustafa Siddique, Jiangying Zeng, Marie E Robert, Haddon Pantel, Anne Mongiu

**Affiliations:** Maimonides Medical Center, Brooklyn, NY, United States; Yale University School of Medicine, New Haven, CT, United States; Maimonides Medical Center, Brooklyn, NY, United States; Maimonides Medical Center, Brooklyn, NY, United States; Yale University School of Medicine, New Haven, CT, United States; Yale University School of Medicine, New Haven, CT, United States; Yale University School of Medicine, New Haven, CT, United States

**Keywords:** schwannoma, mesenchymal tumors, spindle cell tumor, colon mass

## Abstract

Schwannomas of the gastrointestinal tract are rare spindle cell tumors that account for 2–6% of mesenchymal tumors. An elderly male was found to have a left colon mass on CT scan and colonoscopy with pathology of fibrotic tissue. A laparoscopic-assisted left hemi-colectomy with primary anastomosis was performed. Pathology demonstrated spindle cell neoplasm arranged in short fascicles that were strongly and diffusely positive for S100. An elderly female was found to have a submucosal lesion on surveillance colonoscopy in the proximal transverse colon. Biopsy with jumbo forceps revealed spindle cell neoplasm positive for S100. Patient underwent an uncomplicated limited non-oncologic segmental transverse colectomy. We report only the ninth case of left and sixth in the transverse colon described in the literature. As is true for other mesenchymal tumors, mucosal biopsy is usually inconclusive and deep biopsy or submucosal resection is required, making pre-operative surgical decision difficult.

## Introduction

Schwannomas of the gastrointestinal (GI) tract are rare spindle cell tumors that originate from Auerbach’s myenteric plexus [1]. The majority of the time, they are asymptomatic and benign. They can also present with symptoms of obstruction, bleeding and tenesmus [2]. Schwannomas account for 2–6% of all mesenchymal tumors including gastrointestinal stromal tumors (GISTs), leiomyomas, leiomyosarcomas, neurofibromas, paragangliomas, lipomas, granular cell tumors and glomus tumors [1]. In the GI tract, they most often occur in the stomach (83%), small bowel (12%) and least frequently in the colon and rectum [1]. More specifically, in terms of colorectal location, schwannomas occur most frequently in the right colon and cecum (30.2%), the sigmoid colon (28.1%), rectum (21%), the left colon (8.3%) and transverse colon (5.2%) [2]. We describe the cases of patients found to have a schwannoma of the left and transverse colon.

## Case reports

### Case 1

**Figure 1:**
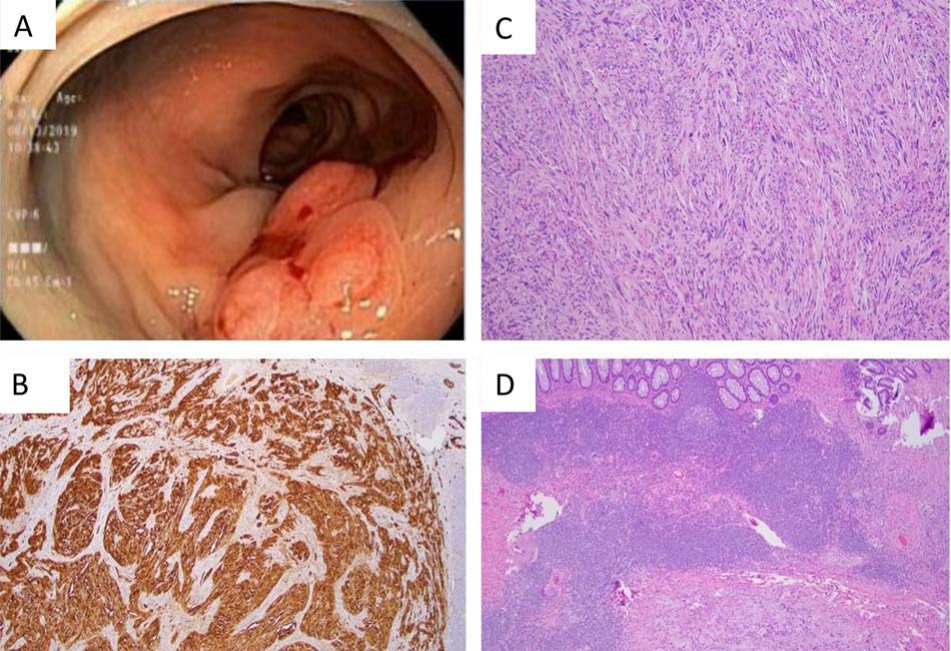
(A) Flat lesion (25 mm) with central depression in the proximal descending colon as seen on colonoscopy. (B) S100 stain 40× demonstrating sheets of strongly S100 positive cells. (C) H&E 100× spindle cells arranged in short fascicles with whirling pattern. (D) H&E 40× demonstrating prominent lymphoid cuff and diffuse lymphoid invasion.

A 60-year-old male with a medical history of congestive heart failure (CHF), hypertension, diabetes mellitus, cerebral vascular event, chronic anemia, and peripheral vascular disease with left digit amputation presented to our medical service with shortness of breath secondary to CHF exacerbation. He received a CT scan and underwent a colonoscopy as work up for chronic anemia, which demonstrated a mass in the left colon. Colonoscopy with biopsy of the area then demonstrated a 25 mm flat lesion with central depression in the proximal descending colon as seen in Fig. 1A. Due to insufficient tissue from biopsy, an additional attempt was made to take a sufficient sample of the polyp with hot snare. Pathology demonstrated fibrotic tissue and no evidence of cancer.

CEA was noted to be mildly elevated at 4.8, his anemia persisted and he required ongoing transfusions. After discussion with a multidisciplinary team, the decision was made to resect the mass. The patient underwent laparoscopic-assisted left hemi-colectomy with primary anastomosis. The patient tolerated the procedure and there were no complications. Post operatively he did well, was placed on ERAS protocol and progressed on diet as tolerated.

Surgical pathology demonstrated a spindle cell neoplasm with cytological atypia as seen in Fig. 1. The tumor measured 2.4 × 1.6 × 0.4 cm and was mainly located in the muscularis propria with extension to serosa. No lymphovascular invasion was seen and 15 lymph nodes were negative for tumor (0/15). Histologically, the specimen revealed a spindle cell neoplasm arranged in short fascicles, nests or a vague whirling pattern. Immunohistochemical stains demonstrated the tumor cells were strongly and diffusely positive for S100 and negative for collagen type 4, melan A, HMB45, AE1/AE3, EMA, DOG-1, c-kit (CD117), MDM2, desmin and SMA. Ki67 showed a proliferative rate of about 3%.

### Case 2

**Figure 2:**
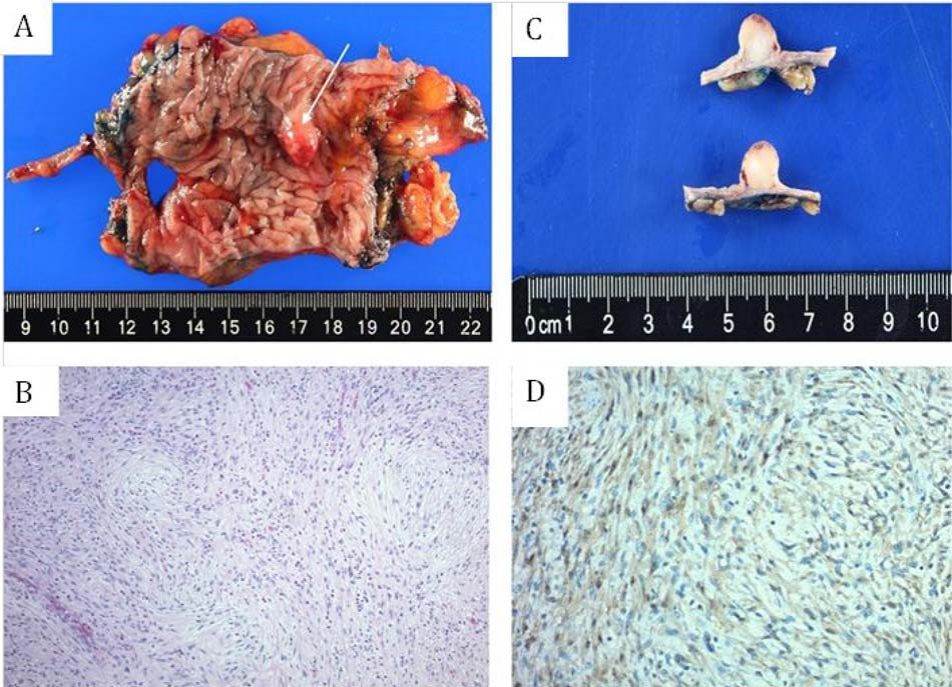
(A and C) gross surgical pathology. (B) H&E staining of spindle cells arranged in short fascicles with whirling pattern. (D) S100 staining demonstrating sheets of strongly S100 positive cells.

A 69-year-old woman with personal history of adenomatous colonic polyps presented for an abnormal finding after undergoing surveillance colonoscopy. Colonoscopy demonstrated a 1-cm submucosal lesion that was found in the proximal transverse colon. Attempts of submucosal injection and snare were not successful after multiple attempts. Successful biopsy was obtained using cold jumbo forceps, which revealed spindle cells positive for S100 consistent with schwannoma. She subsequently underwent an uncomplicated resection that was limited to a non-oncologic segmental transverse colectomy and recovered well postoperatively without issue. Gross and histologic pathology are presented in Fig. 2.

## Discussion

Schwannomas are rare tumors that only account for 2–6% of all mesenchymal tumors [1]. When they occur in the GI tract, they occur with the least frequency in the colon and the rectum. More so, only eight (8.3%) of the reported cases of colorectal schwannomas have been described in the left colon and five (5.2%) in the transverse colon [2]. Here, we have described a case of a 60-year-old male with a schwannoma of his left colon and a 69-year-old female with a schwannoma of her transverse colon.

Schwannomas are usually discovered incidentally during routine screening colonoscopy, but patients have also presented with obstruction, bleeding and tenesmus [2]. When discovered, they are usually diagnosed as a submucosal mass or polyp, with a smooth surface, but can rarely ulcerate into the mucosa [2]. Similarly, the patients reported in this small series presented with history of a GI bleed and anemia and incidentally on surveillance colonoscopy.

Definitive management of schwannoma is the complete surgical resection with free negative margins [2]. Considering the natural history of schwannoma as primarily being a benign tumor (benign in >98% of reported cases), radical surgical resection is not required [2]. When preoperative diagnosis has been made, lesions were resected either endoscopically or by segmental non-oncologic segmental resection [3, 4]. As is true for other mesenchymal tumors, mucosal biopsy is usually inconclusive and deep biopsy or submucosal resection is required, making the pre-operative surgical decision difficult [2]. As a result, in the reported cases, radical surgery has been performed frequently due to the absence of accurate preoperative diagnosis [2]. This was similarly demonstrated in the first case presented, in which the patient received a left hemi-colectomy because preoperative pathology could not distinguish the lesion. In contrast, a preoperative biopsy was obtained for the second patient presented in this small series and a non-oncologic segmental resection was performed.

Definitive diagnosis of schwannoma is made on immunohistochemical pathologic examination in the operative specimen. Tumors are highly cellular and composed of spindle cells often bundled as small clusters in a trabecular pattern with alternating streaks of fibrovascular septa often containing lymphoplasmacytic infiltration [5, 6]. The typical histologic features include a prominent lymphoid cuff and diffuse lymphoid invasion as seen in Fig. 1D. They are composed of sheets of strongly S100 positive cells and occasionally for vimentin, and are negative for SMA, Desmin, CD117 (c-kit) and P53 [6].

The immunohistochemical staining is important in distinguishing schwannoma from other mesenchymal and tumors in the differential diagnosis. Schwannoma differs from GIST as it is consistently negative for CD117 (c-kit), CD34 and DOG-1 which are usually positive in GIST [6–9]. In addition, histologically they also differ because schwannomas have prominent lymphoid cuff and diffuse lymphoid infiltration, cellular heterogeneity, focal nuclear atypia and microtrabecular architectural pattern [6]. Leiomyomas have histologic and immunohistochemical features of well-differentiated smooth muscle cells [6]. Inflammatory myofibroblastic tumors are S100 negative and usually occur in the pediatric population and often involve different abdominal sites in a multifocal manner [6]. Metastatic melanoma is also S100 positive, but will be positive for more specific markers such as HMB45, melan A, tyrosinase and microphthalmia transcription factor [10, 11].

As previously stated, colorectal schwannomas are benign in >98% of reported cases [2]. The aggressiveness of the tumor is dependent on the Ki-67 index and mitotic index. Ki-67 is an indicator of malignancy [2, 6]. With a value of > 5% correlating with greater tumor aggressiveness, and a value of > 10% being considered malignant [12]. In addition, a higher risk of metastasis and/or recurrence has been associated with mitotic activity rate > 5 mitoses per high per field and tumor size greater than 5 cm [13].

GI schwannomas are rare tumors, especially so in the left and transverse colon. Here we report only the ninth case of left colon and sixth in the transverse colon described in the literature. Schwannomas are primarily benign lesions but are difficult to diagnose preoperatively as mucosal biopsy is usually inconclusive and deep biopsy or submucosal resection is required. This makes preoperative surgical planning difficult with such lesions when deciding on adequate resection without a preoperative diagnosis. That is why it is important to have an extensive preoperative work up and understand the differential diagnosis when dealing with such lesions.

## Conflict of interest statement

The authors have no conflicts of interest to disclose.

## Funding

This research did not receive any specific grant from funding agencies in the public, commercial, or not-for-profit sectors.

## Author contributions

Study conception and design was performed by A. Mongiu, H. Pantel and V. Gazivoda. Material preparation and data collection were performed by V. Gazivoda, D. Wang, J. Zeng and M. Robert. The first draft of the manuscript was written by V. Gazivoda, and all authors commented on previous versions of the manuscript. All authors read and approved the final manuscript.
